# Intracardiac Echocardiography to Guide Left Atrial Appendage Occlusion: An Update

**DOI:** 10.31083/RCM28189

**Published:** 2025-04-07

**Authors:** Milos Brankovic, Adamantios Tsangaris, Luka Petrovic, Abhishek Sharma

**Affiliations:** ^1^Cardiovascular Division, Department of Medicine, University of Minnesota, Minneapolis, MN 55455, USA; ^2^Department of Cardiology, Icahn School of Medicine, Mount Sinai Fuster Heart Hospital, Mount Sinai Morningside Hospital, New York, NY 10025, USA; ^3^Division of Cardiology, Department of Medicine, Rutgers New Jersey Medical School, Newark, NJ 07103, USA

**Keywords:** left atrial appendage occlusion, LAA closure, intracardiac echocardiography, ICE, transesophageal echocardiography, TEE, atrial fibrillation, stroke, anticoagulation

## Abstract

The left atrial appendage occlusion (LAAO) procedure is an important intervention for stroke prevention in patients with non-valvular atrial fibrillation who cannot tolerate anticoagulation. Accurate imaging is essential to guide and ensure optimal device deployment. Transesophageal echocardiography (TEE) has traditionally been the gold standard for procedural guidance, but intracardiac echocardiography (ICE) is emerging as an alternative owing to its unique advantages. This review examines the comparative effectiveness, procedural advantages, limitations, and clinical outcomes of ICE and TEE in LAAO closure, highlighting emerging trends and implications for future clinical practice.

## 1. Introduction

Intracardiac echocardiography (ICE) is an advanced imaging technique that utilizes ultrasound to visualize the heart’s structures and function from within the cardiac chambers in real-time [1]. Currently, there are two main types of ICE catheters—rotational and phased-array catheters—each with unique features tailored to different applications [2]. Rotational ICE catheters are non-steerable, near-field imaging devices primarily used to help with transeptal puncture in left-sided electrophysiological studies. Conversely, phased-array ICE catheters are steerable and capable of far-field imaging that can create two-dimensional (2D) images like those traditionally obtained by transesophageal echocardiography (TEE) [3]. Phased-array ICE catheters have been used for intra-procedural guidance of left atrial appendage occlusion (LAAO) and other structural interventions [4, 5].

The ICE procedure typically involves the insertion of a specialized ultrasound catheter through the femoral vein, which is then advanced into the right atrium. The ICE catheter can be further positioned into the right ventricle, pulmonary artery, coronary sinus, and after atrial septal puncture into the left atrium. This allows for high-resolution imaging to guide interventions such as LAAO [6, 7, 8], arrhythmia ablation [9, 10], closure of atrial and ventricular septal defects, patent foramen ovale [11, 12, 13], and percutaneous valvular interventions [14, 15]. The ICE is also used to rule out intracardiac thrombus [16], evaluate the anatomy of pulmonary veins [17], guide endomyocardial biopsy [18], and evaluate prosthetic valve or lead infection [19, 20]. As the field of interventional-structural cardiology continues to advance, ICE application is expected to expand and enhance the safety and efficacy of various cardiac procedures.

Despite recent observational studies and meta-analyses showing similar procedural success and overall complication rates of ICE- and TEE-guided LAAO, each imaging modality still carries different implications for procedural guidance. For this reason, it is important to consider patient-specific profiles, operator expertise, and institutional resources when choosing between ICE and TEE for procedural guidance. This narrative review examines the comparative effectiveness, procedural advantages, limitations, and clinical outcomes of ICE and TEE in LAAO, highlighting emerging trends and implications for clinical practice.

## 2. LAAO for Stroke Prevention

The LAAO procedure has been approved for stroke prevention in patients with non-valvular atrial fibrillation (AF) and high risk of stroke as an alternative to anticoagulation. In the United States, the Food and Drug Administration (FDA) approved the Watchman device (Boston Scientific) in 2015 based on the results of PROTECT-AF [21] and PREVAIL [22] trials, which showed its safety and non-inferiority compared to warfarin. Subsequently, the Amulet IDE [23] trial showed the non-inferiority of the Amplatzer Amulet device (Abbott) compared to the Watchman device, leading to its FDA approval in 2021. Likewise, the PRAGUE-17 trial demonstrated non-inferiority of LAAO (Watchman/Watchman-FLX or Amulet) compared to direct oral anticoagulation agents in preventing major AF-related cardiovascular, neurological, and bleeding events [24, 25].

Current 2023 American College of Cardiology / American Heart Association / American College of Chest Physicians / Heart Rhythm Society (ACC/AHA/ACCP/HRS) guidelines recommend the use of LAAO in non-valvular AF patients with at least a moderate risk of stroke (CHA_2_DS_2_VASc score ≥2) and contraindication for long-term oral anticoagulation due to non-reversible cause (class 2a), or in those at high risk of major bleeding on oral anticoagulation (class 2b) [26]. The latest Society for Cardiovascular Angiography and Interventions/Heart Rhythm Society (SCAI/HRS) Expert consensus statement on transcatheter LAAO also endorses these recommendations and provides additional guidance on institutional and operator requirements for LAAO procedure [5].

## 3. ICE Technology for LAAO

Traditionally, TEE has been the gold standard imaging modality for ruling out left atrial appendage (LAA) thrombus and ensuring proper selection, size, deployment, and stability of LAAO devices. However, ICE has gained popularity as an alternative due to its cost-effectiveness. It does not require an extra operator, requires less general anesthesia and endotracheal intubation [6, 27], shorter procedural time [28, 29], and less radiation exposure [30, 31]. These clinical benefits of ICE have provided reassurance about its practicality and affordability. Nonetheless, ICE placement is an invasive procedure that requires venous access and occasionally an additional transeptal puncture with an increased risk of iatrogenic atrial septal defect (iASD) and pericardial effusion [6, 27]. It also has a smaller field of view than a TEE, though recent advancements address this limitation [32, 33].

As a single-plane imaging modality, a 2D ICE might be suboptimal for precise landing zone assessment and device sizing as LAA exhibits variability in orientation, size, shape, and number of lobes. This shortcoming of 2D ICE catheters can be overcome by combining intra-procedural ICE imaging with pre-procedural coronary computed tomography angiography (CCTA) with three-dimensional (3D) reconstruction or TEE during pre-procedural planning [34, 35]. The pre-procedural planning includes ruling out the presence of intracardiac thrombus, assessing LAA anatomy, measuring the plane of maximum landing zone diameter, and device sizing.

There are now commercially available phased-array ICE catheters with 3D capabilities, such as a 12.5 Fr 90 cm AcuNav Volume 3D ICE catheter (Siemens Healthineers), a 10 Fr 90 cm NuVision 3D ICE catheter (Biosense Webster), and a 9 Fr 90 cm VeriSight Pro 3D ICE catheter (Philips). These 3D ICE catheters are superior to 2D catheters due to their accurate volumetric display, improved spatial resolution, and superior Doppler analysis of peri-device leak following device deployment [32, 33]. They have a high level of agreement with pre-procedural TEE for LAA sizing and were found to be superior to 2D-ICE for device sizing [32].

## 4. ICE-Guided LAAO Procedure

The main advantage of ICE over TEE is for intra-procedural LAAO guidance. Specifically, it involves real-time ICE imaging to guide trans-septal puncture, device positioning and deployment, assessment of device stability, peri-device leak, and immediate complications. Pericardial effusion, thrombus formation on the catheter or LAAO device, residual iASD and air embolism are immediate complications easily identified by ICE. Imaging of the pericardial space should be performed at the beginning and end of the procedure to assess for pericardial effusion.

**Fig. 1.**
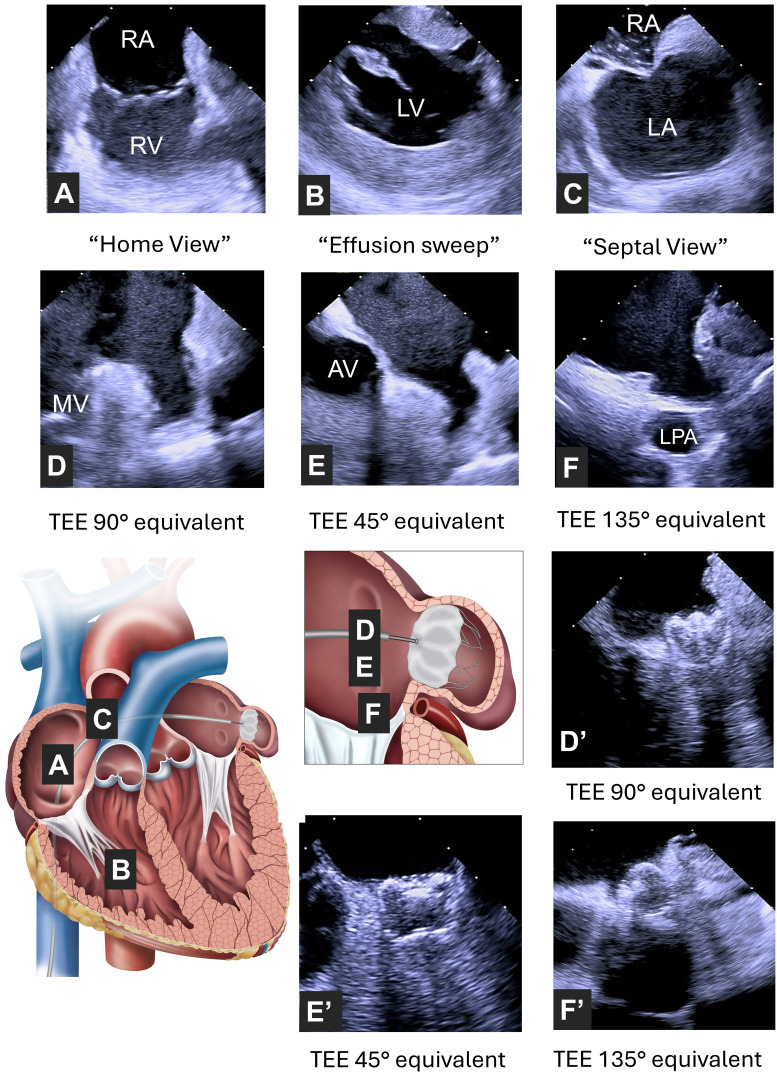
**Illustration of the imaged structures with an ICE probe during a left atrial appendage occlusion procedure**. (A) A “home view” of the long axis of the right atrium and ventricle separated by the tricuspid valve. (B) A pericardial effusion sweep is performed from the right ventricle to rule out pericardial effusion before performing the transeptal puncture. (C) The ICE catheter captures a “septal view” of interatrial septum with tenting by the needle ready to cross; ICE catheter in retroflex mid-LA position with MV in view (equivalent to TEE 90° view) pre-deployment (D) and post-deployment phase (D’). ICE catheter from retroflex mid-LA position with AV in view (equivalent to TEE 45° view) pre- deployment (E) and post-deployment phase (E’). ICE catheter in supra-mitral LA position with LPA in view (equivalent to TEE 135° view) pre- deployment (F) and post-deployment phase (F’). AV, aortic valve; MV, mitral valve; LA, left atrium; LV, left ventricle; LPA, left pulmonary artery; RA, right atrium; RV, right ventricle; ICE, intracardiac echocardiography; TEE, transesophageal echocardiography.

Here’s an overview of the general steps for ICE imaging of the LAA. Briefly, the ICE catheter is advanced over the guidewire via venous femoral access to the right atrium through the inferior vena cava under fluoroscopy. Once in the right atrium, ICE imaging starts by capturing a “home view” of the long axis of the right atrium and ventricle separated by the tricuspid valve (Fig. 1A, Video 1). A pericardial effusion sweep is performed from the right atrium or right ventricle to rule out pericardial effusion before performing the transeptal puncture (Fig. 1B, Video 1). The LAA is interrogated from the right atrium, right ventricle, and/or the pulmonary artery in a preliminary attempt to assess for thrombus.

**Video 1.** **The ICE catheter in the right atrium “home view” and effusion sweep from the right atrium and right ventricle**. ICE, intracardiac echocardiograph. Video associated with this article can be found, in the online version, at https://doi.org/10.31083/RCM28189.

Back from the “home view”, further clockwise rotation and slight retraction or advancement of the ICE catheter captures a “septal view”, equivalent to the TEE bi-caval view (Fig. 1C, Video 2). Using a biplane mode, 3D ICE catheters provide clear imaging of the interatrial septum for simultaneous orientation in superior-inferior and anterior-posterior axes to access the left atrium. The ICE catheter is usually delivered to the left atrium through a single puncture with the LAAO device but occasionally can require an additional transeptal puncture.

**Video 2.** **The ICE catheter in the right atrium “septal view” capturing transeptal puncture**. ICE, intracardiac echocardiography. Video associated with this article can be found, in the online version, at https://doi.org/10.31083/RCM28189.

Once the left atrium is reached, the ICE catheter is directed through the septum into the left atrium to assess for thrombus and confirm LAA measurements. For sizing of non-lobe-and-disc occluder devices, the LAA ostium is measured from the circumflex artery to the point 2 mm below the tip of the left upper pulmonary vein limbus. When placing lobe-and-disc occluders, the ostium is measured from the top of the mitral valve annulus to 2 mm from the tip of the pulmonary vein limbus [36]. The ICE catheter provides continuous imaging during the procedure, ensuring accurate positioning and deployment of the LAAO device.

The LAA is usually evaluated in three ICE positions: retroflex mid-LA position with the mitral valve in view (equivalent to TEE 90° view (Fig. 1D, Video 3)), retroflex mid left atrial position with rightward tilt and counter-clockwise rotation to bring the aortic valve in view (equivalent to TEE 45° view (Fig. 1E, Video 4)), and supra-mitral LA position with the left pulmonary artery in view (equivalent to TEE 135° view (Fig. 1F, Video 5)). An additional view is sometimes required by placing the ICE catheter in the left upper pulmonary vein (LUPV), which can help to better appreciate LA depth or if there is a need for better catheter stability. The ICE probe in the LUPV can provide TEE equivalent views from 0–90° with rightward or leftward deflection, respectively. When using 3D ICE imaging, multiplanar reconstruction of the appendage allows the assessment of the landing zone’s maximum and minimum diameters and depth for more precise device sizing.

**Video 3.** **The ICE catheter in the left atrium TEE 90° equivalent view**. ICE, intracardiac echocardiography; TEE, transesophageal echocardiography. Video associated with this article can be found, in the online version, at https://doi.org/10.31083/RCM28189.

**Video 4.** **The ICE catheter in the left atrium TEE 45° equivalent view**. ICE, intracardiac echocardiography; TEE, transesophageal echocardiography. Video associated with this article can be found, in the online version, at https://doi.org/10.31083/RCM28189.

**Video 5.** **The ICE catheter in the left atrium TEE 135° equivalent view**. ICE, intracardiac echocardiography; TEE, transesophageal echocardiography. Video associated with this article can be found, in the online version, at https://doi.org/10.31083/RCM28189.

After device deployment, the ICE confirms proper seating, anchoring, sizing, and the absence of residual peri-device leak and pericardial effusion. The ICE catheter is then carefully retracted, ensuring there is no trauma to cardiac structures. The presence of an interatrial shunt is assessed from the right atrium (Video 6).

**Video 6.** **The ICE catheter in the right atrium “septal view” to assess residual interatrial shunt**. ICE, intracardiac echocardiography. Video associated with this article can be found, in the online version, at https://doi.org/10.31083/RCM28189.

## 5. Effectiveness and Safety of ICE and TEE

The ICE LAA was the first study to prospectively evaluate the effectiveness and safety of ICE-guided Watchman FLX implantation in 100 patients undergoing LAAO [7]. The authors demonstrated 100% procedural success rates and no conversion to TEE or the presence of pericardial effusion, peri-device leak, device embolization, and device-related thrombosis at 45-day follow-up. More recently, the National Cardiovascular Data Registry (NCDR) LAAO Registry compared 2272 ICE-guided LAAO cases and 31,835 TEE-guided cases, demonstrating similar success rates of device implantation between ICE and TEE (ICE 98.3% versus TEE 97.6%) [6]. There was no difference in the rates of a complete seal (ICE 83% versus TEE 82%) and mortality (ICE 1.1% versus TEE 0.8%) at 45 days. A recently updated meta-analysis of 19 observational studies with 4415 ICE-guided cases and 38,059 TEE-guided cases supports these findings by showing comparable overall complication rates [27]. This meta-analysis also showed that ICE-guided LAAO cases had 33% higher odds of procedural success than TEE-guided cases. Given the observational design of included studies, it remains unclear whether these estimates are affected by selection bias. Nonetheless, studies suggest that both methods achieve comparable high success rates and a high overall safety profile.

When analyzing individual safety endpoints, ICE had on average 2-fold higher odds of pericardial effusion compared to TEE at 45 days [27]. These rates are influenced by the study regions and the operator’s ICE experience [6]. It remains unclear if the pericardial effusion is related to trans-septal puncture, device deployment, or manipulation of the ICE catheter. Despite the increased relative risk of pericardial effusion, it is important to note the absolute rates of ICE-related pericardial effusion are low, ranging from 0.5 to 1% at 45 days [6]. Similarly, ICE has been linked to higher rates of immediate residual iASD compared to TEE [27]. However, current studies have suggested a high closure rate of iASD during follow-up and no significant adverse clinical events [37, 38]. Complications such as pericardial effusion and iASD are relatively higher for ICE than for TEE but they can be further reduced by increasing the operator’s ICE experience.

Notably, the NCDR LAAO Registry has shown that patients who underwent ICE-guided LAAO had a 40% relative risk reduction for general anesthesia and a 20% higher chance of same-day discharge than TEE. The TEE-guided LAAO often requires deep sedation because of the transesophageal probe. In contrast, ICE imaging is generally performed under conscious sedation or mild sedation, which shortens recovery and is preferable for high-risk patients. The benefit of avoiding general anesthesia is of particular importance for patients undergoing LAAO as they are typically frail with multiple comorbidities [39]. Similarly, studies have shown reduced turnover time in the catheterization lab and contrast use when using ICE to guide LAAO [29, 40].

## 6. Cost Implications of ICE

The insurance reimbursement for the ICE system can vary based on several factors, such as type of insurance provider, Current Procedural Terminology (CPT) codes and modifiers, medical necessity, and prior authorization. Private insurance companies, Medicare, and Medicaid each have reimbursement policies, with rates and conditions that may differ by region and setting (e.g., hospital outpatient or inpatient). ICE’s procedure code (CPT) is typically 93662, designated for intracardiac echocardiography. Insurance plans may require proof of medical necessity and sometimes prior authorization for reimbursement. Although ICE catheters are expensive, the overall procedural cost difference between ICE and TEE-guided LAA closure is complex. Savings from reduced anesthesia requirements, shorter recovery times, and fewer staff requirements offset ICE’s higher initial costs [28, 30]. Finally, ICE may be necessary for complex procedures and transseptal punctures where real-time imaging significantly improves procedural outcomes and patient safety.

## 7. Conclusions

Both ICE and TEE have strengths and limitations in guiding LAA closure. Advancements in ICE imaging technology are expected to enhance ICE’s utility in LAAO procedures. Ongoing studies compare clinical outcomes, cost-effectiveness, and procedural times and will provide further insight into optimal imaging practices for LAAO. The TEE remains advantageous for comprehensive visualization and has an established role in structural heart procedures. However, ICE offers significant advantages in patient comfort, especially for patients unable to tolerate general anesthesia and when esophageal or endotracheal intubation is contraindicated. Proper imaging modalities should be selected based on patient-specific factors, operator expertise, and institutional resources. Future research will help clarify which modality offers superior outcomes for specific patient populations.
